# Striatonigral direct pathway 2-arachidonoylglycerol contributes to ethanol effects on synaptic transmission and behavior

**DOI:** 10.1038/s41386-023-01671-8

**Published:** 2023-08-01

**Authors:** Shana M. Augustin, Alexa L. Gracias, Guoxiang Luo, Rishitha C. Anumola, David M. Lovinger

**Affiliations:** 1 Laboratory for Integrative Neuroscience, National Institute on Alcohol Abuse and Alcoholism, National Institutes of Health, Bethesda, MD 20892 USA; 2 Department of Pharmacology, Northwestern University Feinberg School of Medicine, Chicago, IL 60611 USA; 3 Department of Psychiatry and Behavioral Sciences, Northwestern University Feinberg School of Medicine, Chicago, IL 60611 USA

**Keywords:** Reward, Addiction

## Abstract

Endocannabinoids (eCB) and cannabinoid receptor 1 (CB1) play important roles in mediating short- and long-term synaptic plasticity in many brain regions involved in learning and memory, as well as the reinforcing effects of misused substances. Ethanol-induced plasticity and neuroadaptations predominantly occur in striatal direct pathway projecting medium spiny neurons (dMSNs). It is hypothesized that alterations in eCB neuromodulation may be involved. Recent work has implicated a role of eCB 2-arachidonoylglycerol (2-AG) in the rewarding effects of ethanol. However, there is insufficient research to answer which cellular subtype is responsible for mediating the 2-AG eCB signal that might be involved in the rewarding properties of ethanol and the mechanisms by which that occurs. To examine the role of dMSN mediated 2-AG signaling in ethanol related synaptic transmission and behaviors, we used conditional knockout mice in which the 2-AG-synthesizing enzyme diacylglycerol lipase α (DGLα) was deleted in dMSNs, DGLα^D1-Cre+^. Using acute brain slice photometry and a genetically encoded fluorescent eCB sensor, GRAB_eCB2.0,_ to assess real-time eCB mediated activity of sensorimotor inputs from primary motor cortices (M1/M2) to the dorsolateral striatum, we showed that DGLα^D1-Cre+^ mice had blunted evoked eCB-mediated presynaptic eCB signaling compared to littermate controls. Furthermore, ethanol induced eCB inhibition was significantly reduced in DGLα^D1-Cre+^ deficient mice. Additionally, there was a reduction in the duration of loss of righting reflex (LORR) to a high dose of ethanol in the DGLα^D1-Cre+^ mice compared to controls. These mice also showed a male-specific decrease in ethanol preference accompanied by an increase in ethanol-induced water consumption in a voluntary drinking paradigm. There were no significant differences observed in sucrose and quinine consumption between the genotypes. These findings reveal a novel role for dMSN mediated 2-AG signaling in modulating ethanol effects on presynaptic function and behavior.

## Introduction

Alcohol use is widespread, with 219.2 million people aged 12 or older reporting use [1]. Undoubtingly, alcohol misuse which can potentially result in Alcohol Use Disorder (AUD) is a public health concern. Despite this concern, much remains to be determined about the neurobiological underpinnings contributing to alcohol misuse and the pathological progression of AUD.

The endocannabinoid (eCB) system has been implicated in the reward circuitry that reinforces the addictive effects of misused substancess [2–5]. In the brain, the main types of eCBs are N-arachidonoylethanolamine (anandamide or AEA) and 2-arachidonoylglycerol (2-AG). These lipid-derived molecules exert their neural functions via CB1 and CB2 receptors which are found throughout the brain [6, 7]. The primary projection neurons in the striatum, medium spiny neurons (MSNs) categorized into two types, striatopallidal indirect-projecting pathway MSNs (iMSNs) and striatonigral direct-projecting pathway MSNs (dMSNs), can synthesize and release eCBs to then bind to presynaptic CB1 receptors [3, 8–10]. This can result in the regulation of striatum mediated behaviors, such as goal-directed, habitual, and reward-driven behaviors through synaptic plasticity involving eCBs [3, 11, 12]. Striatal MSNs receive dense glutamatergic projections from several brain areas, such as the cortex, hippocampus, and thalamus [13–15]. These projections, particularly from the cortex, play a significant role in modulating striatal output involved in motor functions, as well as the reinforcement of motor behaviors [16–21]. Strengthening of sensorimotor cortical projections to dorsolateral striatum (DLS) appears to contribute to the progression from goal-directed to habitual drug seeking/taking. However, the role of eCBs at specific corticostriatal projections remains unclear.

Endocannabinoids and CB1 receptors are involved in ethanol actions in the brain [22–24]. CB1 receptor antagonism decreases ethanol consumption [25–30], indicating that eCB signaling may be important for the reinforcement of alcohol seeking and taking behaviors. Furthermore, CB1 receptor deletion results in reduced ethanol self-administration and place-preference [31, 32]. Striatal CB1 receptor dependent long-term depression is also impaired following ethanol exposure [33, 34]. There is mounting evidence suggesting that ethanol exposure disproportionately affects striatal dMSN synaptic signaling and structural morphology [35]. Indeed, ethanol disrupts synaptic transmission at dMSN synapses [36, 37]. Moreover, ethanol exposure increases dMSN dendritic length, presumably increasing the synaptic contacts made onto spines [35, 38]. This is a possible mechanism by which ethanol consumption can strengthen glutamatergic input signaling onto dMSNs [34, 35]. Furthermore, activation of striatal dMSNs facilitates ethanol consumption [34]. Self-administration of ethanol increases striatal dialysate 2-AG, and not AEA concentrations [39]. Moreover, global deletion of brain diacylglycerol lipase α (DGLα), the synthesizing enzyme for 2-AG, reduces EtOH consumption [40]. Although the roles of CB1 receptors and 2-AG in ethanol related behaviors have been examined, the precise mechanisms and location of eCB, specifically the role of 2-AG in mediating ethanol-induced synaptic alterations, remains unknown. Herein, we elucidated ethanol effects on dMSN-derived 2-AG and the role of this signaling in ethanol-induced behaviors.

## Materials and methods

Experiments were approved by the National Institute on Alcohol Abuse and Alcoholism Animal Care and Use Committee and were carried out in accordance with NIH guidelines. Cell-specific dMSN DGLα floxed mice (DGLα^flx/flx^) were obtained from Vanderbilt University [41] and bred in-house. Experiments were conducted during the light phase, unless otherwise stated. Animals were housed on a 12 h light-dark cycle with food and water ad libitum. Both sexes (9–18 wks old) were used for experiments.

### Generation of dMSN DGLα Knockout (DGLα^D1-Cre+^) mice

Transgenic DGLα^D1-Cre+^ mice were generated as previously described [41]. Briefly, mice containing loxP sites flanking exon 9 of the endogenous Dagla gene, DGLα ^flx/flx^ were crossed with the Drd1 Cre (D1-Cre; GENSAT Project; MGI ID:3836633) mice to generate DGLα^D1-Cre+^ and littermate controls, DGLα^flx/flx^.

### Stereotaxic intracranial injection

Mice underwent bilateral stereotaxic intracranial surgery under isoflurane anesthesia. For slice photometry, DGLα^D1-Cre+^ and DGLα^flx/flx^ mice were injected with 250 nL of a G-protein-coupled receptor (GPCR) activation-based, GRAB_eCB2.0_ sensor adeno-associated virus vector (AAV) *AAV9-hSYn.-eCB 2.0* (1 × 10^13^GC/mL) or GRAB_eCBmut_ sensor (non-binding eCB sensor used as a negative control) *AV9 AAV9-hSyn-eCB 2.0-mut* (1 × 10^13^GC/mL; ViGene Biosciences) at a rate of 50nL/min into primary motor cortex (from Bregma; AP: 1.0 mm, ML ±1.7 mm, from brain surface DV −0.9 mm). Slice photometry experiments were performed at least 4 weeks after viral injection using techniques described by Liput et al., (2022) [42].

### Brain slice preparation

For slice photometric recordings, mice were anaesthetized with isoflurane and immediately decapitated. Brains were removed and placed in oxygenated (95% O_2_ and 5% CO_2_), ice cold sucrose containing artificial cerebrospinal fluid (aCSF) containing (in mM): 194 sucrose, 30 NaCl, 4.5 KCl, 1 MgCl_2_, 26 NaHCO_3_, 1.2 NaH_2_PO_4_, 10 D-glucose. Coronal brain slices (250 µm) containing striatum were prepared using a vibratome (LEICA—VT1200S). Slices were then incubated at 32 °C for 30 min in oxygenated-aCSF containing in mM: 124 NaCl, 4.5 KCl, 1 MgCl_2_, 26 NaHCO_3_, 1.2 NaH_2_PO_4_, 10 D-glucose, and 2 CaCl_2_ prior to recordings.

### Brain slice photometry

Photometric recordings were acquired from eCB2.0 and eCBmut GFP-expressing brain hemisections. Slices were perfused with oxygenated aCSF at 1–2 ml/min at 30–32 °C.  A twisted bipolar stimulating electrode (Plastics One) was placed in the DLS outside the fluorescence excitation window (180µm^2^). Single pulse stimuli (0.6 mA, 2 ms) were generated using a current stimulator (DS3, Digitimer Ltd, Hertfordshire, UK). Short trains of stimuli (5 pulses (Ps), 5 Hz; 10Ps, 10 Hz; 20Ps, 20 Hz; 50Ps, 50 Hz; 100Ps, 100 Hz) were generated by a Master 8 stimulator (A.M.P.I., Jerusalem, Israel). Fluorescence emission was collected using a 40x/0.8NA water immersion objective on an upright fixed stage Olympus BX41 microscope (Olympus Life Science Solutions, Waltham, MA, USA). Fluorescence from the GRAB_eCB2.0 or mut_ sensor containing a circular permuted GFP moiety was excited using 470 nm light emitted from an LED (Thorlabs, Newton, NY, USA) every 3 min in 1 min and 20 s intervals. A shutter (uniblitz), triggered by pCLAMP software (Molecular Devices, CA, USA 11.03) was used to minimize exposure time and photobleaching. Emitted light was filtered with a FITC filter set (Ex 470/40, FT 495, Em 525/50) and directed to a photomultiplier tube (PMT; Model D-104, Photon Technology International, Edison, NJ, USA). The PMT voltage output (time constant: 5 ms; gain: 1 µA) was filtered at 1 kHz and digitized at 10 kHz using Digidata 1500B (Molecular Devices) and Clampex software (Axon instruments). The data were analyzed using pCLAMP software. Evoked eCB transients were measured as the ratio of the evoked peak fluorescence amplitude of the eCB2.0 or eCBmut transient (ΔF) to the average baseline (5 sweeps, 15 mins) fluorescence value (F) before stimulation, generating a ΔF/F value. The data are expressed as percent change of ΔF/F relative to baseline.

### Loss of the righting-reflex (LORR)

To assess the sedative effect of ethanol by measuring the latency and duration of LORR, mice were administered 3.5 g/kg ethanol intraperitoneally (i.p.).  LORR latency was defined as the time to lose righting-reflex, which was determined as the time from injection to the inability of the mouse to right itself within 30 s of being placed in a supine position in a V-shaped plexiglass trough. The duration of LORR was defined as the time between losing and regaining the righting-reflex. Mice were determined to have regained their reflex if they were able to right themselves 3 times within 30 s.

### Two-bottle choice 16 h brief ethanol intermittent access (bIA)

Mice were singly housed and allowed to habituate to the two-bottle choice cages for 8–12 days for ethanol intake assessment. During this period, both drinking bottles contained water. For the two-bottle choice ethanol bIA (3 times a week, every-other-day), the home cage water bottles were replaced with one bottle containing ethanol and the other water 1 h prior to lights-off. To prevent side preferences, the position of the bottles was rotated. Mice were given access to increasing concentrations of ethanol (5%, 10% & 20% v/v with water) successively on a weekly basis (1wk /concentration) for a total of 3 weeks. Bottles containing ethanol and water were weighed before and after drinking sessions. On non-ethanol drinking days, mice had free access to water. Differences in weights of the bottles were converted to grams of ethanol consumed per kilogram of body weight. The preference for ethanol was determined as the volume of ethanol consumed over the total fluid (water and ethanol) consumed. Data were collected from two separate cohorts of mice.

### Ethanol challenge injection

Mice singly housed with prior exposure to ethanol (LORR testing) were given an i.p. injection of 3.5 g/kg ethanol an hour before the dark cycle. For consistency, we used the same dose of ethanol used for LORR testing. The amount of water consumed after 16 hrs of overnight drinking was measured and the grams of water consumed per kilogram of body weight calculated.

### Sucrose & quinine consumption

Mice previously exposed to ethanol were given 16 h access to either a bottle containing sucrose or quinine solution for 6 consecutive days each accompanied with a bottle containing water to determine taste preference. Mice were tested on three ascending concentrations of sucrose, 0.5%, 2%, and 10%, as well as three quinine concentrations, 0.03 mM, 0.10 mM, and 1 mM for 2 days each. Sucrose consumption was measured 2 weeks prior to quinine consumption. Solution preference was determined as the ratio of a given solution intake relative to the total fluid intake. Ratios were averaged across the 2 days.

### Water restriction

Following quinine consumption, singly housed mice were habituated to a single water bottle cage for one week. The water bottle was removed overnight and returned after 16 h of water deprivation. The water bottle was weighed after 8 h to determine consumption.

### Drugs

DO34—3-(Phenylmethyl)−4-[[4-[4-(trifluoromethoxy) phenyl]−1H-1,2,3-triazol-1-yl] carbonyl]−1-piperazinecarboxylic acid 1,1-dimethylethyl ester (Glixx Laboratories Inc, CAS #:1848233-58-8), AM251 (Tocris), alcohol (Sigma, St. Louis, MO), and tetrodotoxin (TTX, Biotium, Fremont, CA) were diluted to their final concentration in aCSF for acute slice recordings. For behavioral experiments, alcohol, sucrose (Sigma, St. Louis, MO), and quinine (Alfa Aesar, Tewksbury, MA) were diluted in water.

### Statistical analyses

Data were analyzed using unpaired two-tailed *t* test and a one-, two-, or three-way ANOVA with repeated measures (rmANOVA) in GraphPad Prism. For alcohol intake, data were analyzed by three-way rmANOVA in SPSS. Data are reported as mean ± SEM.

## Results

### Real-time 2-AG signaling disruption in dMSN DGLα KO mice

**Fig. 1:**
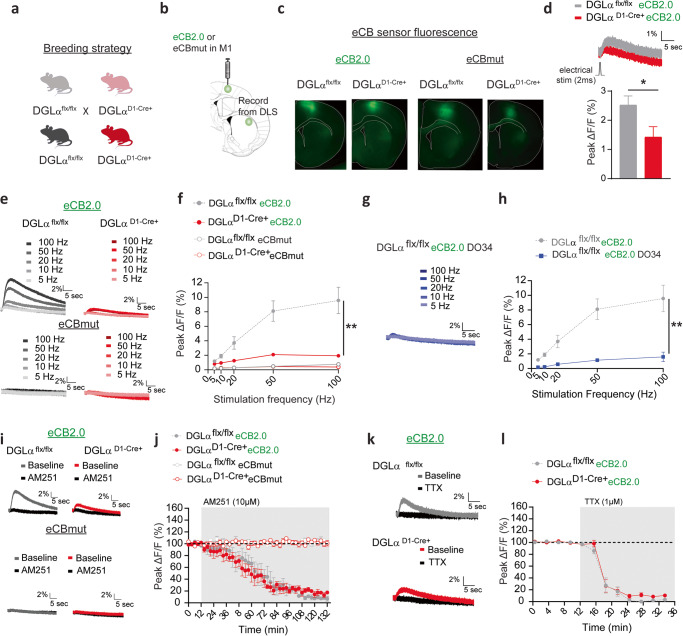
GRAB_eCB2.0_ detects 2-AG signaling deficits in dMSN DGLα KO mice. **a** Schematic of breeding strategy. **b** Diagram showing viral eCB2.0 injections into M1 and recording site in the DLS. **c** eCB2.0 immunoreactivity in M1 somata and DLS inputs. **d** *Top*. Representative traces of normalized eCB transient evoked by a single electrical pulse. Bottom. Averaged peak amplitude. **e** Representative traces of eCB transients evoked by a train of electrical stimulation. **f** Averaged Peak amplitude of eCB transients as a function of stimulation frequency and number of pulses. **g** Representative traces and **h** averaged peak amplitude of eCB transients evoked by train electrical stimulation after preincubation with D034. **i** Representative traces before and after CB1 receptor antagonist, AM251. **j** Evoked eCB transients in the presence of AM251. **k** Representative traces before and after bath application of sodium channel blocker, TTX. **l** Evoked eCB transients in the presence of TTX. Data shown as the mean ± SEM. *p < 0.05, **p < 0.01.

Previous work from our laboratory showed that 2-AG increases are primarily evoked with brief striatal stimulation measured in corticostriatal terminals of C57Bl6J mice [42]. To confirm if striatal eCB mobilization was disrupted in mice in which the 2-AG synthesizing enzyme DGLα was disrupted in dMSNs (Fig. 1a), we used acute brain slice photometry and the newly engineered CB1-receptor derived optical eCB, GRAB_eCB2.0_ (eCB 2.0) or null mutant, GRAB_mut_ (eCBmut) sensor [43]. Either the eCB2.0 or eCBmut sensor was injected into motor cortex (M1/M2) to assess evoked presynaptic eCB/GRAB_eCB2.0_-mediated fluorescence transients at corticostriatal terminals in DLS of dMSN 2-AG deficient mice, DGLα^D1-Cre+^, or littermate DGLα^flx/flx^ controls (Fig. 1a–c). There was a reduction in the amplitude of eCB transients in DGLα^D1-Cre+^ mice expressing eCB2.0 evoked by single stimuli (Fig. 1d, DGLα^flx/flx^ 2.5% ± 0.3%, *n* = 7 slices, DGLα^D1-Cre+^ 1.4% ± 0.4% n = 8 slices, unpaired two-tailed *t* test: t_13_ = 2.283, *p* < 0.05,) or stimulus trains (Fig. 1e, f, rmANOVA genotype main effect: f _1,11_ = 14.51, p = 0.003; train stimulation x genotype interaction: F_4,44_ = 12.47, *p* < 0.0001, DGLα^flx/flx^ *n* = 7 slices; DGLα^D1-Cre+^ *n* = 6 slices,) compared to DGLα^flx/flx^ mice expressing eCB2.0. Moreover, evoked eCB transients in slices expressing eCBmut were very small to undetectable in DGLα^D1-Cre+^ and DGLα^flx/flx^ mice with train stimulation measured at corticostriatal afferents and did not differ between genotypes (Fig. 1e, f rmANOVA genotype main effect: f _1,19_ = 0.913, *p* > 0.05, DGLα^flx/flx^ *n* = 11 slices; DGLα^D1-Cre+^ *n* = 10 slices). These findings support previous findings that GRAB_eCB2.0_ detects eCB release in striatal slices and indicate that a substantial component of this signal involves 2-AG release from dMSNs [42].

It was previously shown that preincubation of slices with the diacylglycerol lipase inhibitor DO34 reduces eCB transients detected at corticostriatal afferents [42]. To test whether this inhibitor reduces evoked eCB transients in DGLα^flx/flx^ mice to a similar degree observed in the DGLα^D1-Cre+^, slices from DGLα^flx/flx^ mice were preincubated in DO34 (1uM). Indeed, evoked eCB transients in DGLα^flx/flx^ mice were reduced by DO34 compared to untreated slices (Fig. 1g, h; data from Fig. 1f re-graphed in Fig. 1h for comparison, rmANOVA treatment main effect: f _1,13_ = 21.95, *p* = 0.0004; train stimulation x treatment interaction, F_4,52_ = 13.87, *p* < 0.0001, DGLα^flx/flx^ *n* = 7 slices), consistent with other evidence of 2-AG release [42]. The reduction in the evoked eCB transients resembled that seen in DGLα^D1-Cre+^ mice (Fig. 1f, h; rmANOVA treatment main effect: f _1,12_ = 1.559, *p* > 0.05).

Evoked fluorescent transients were also reduced by application of the CB1 inverse agonist AM251 (10 µM) in DGLα^flx/flx^ and DGLα^D1-Cre+^ eCB2.0 expressing slices compared to baseline, confirming CB1 receptor and eCB specificity (Fig. 1i, j; DGLα^flx/flx^ 8% ± 3%, Mann–Whitney test, two-tailed *p* < 0.05, *n* = 4; DGLα^D1-Cre+^ 16% ± 4%, Mann–Whitney test, two-tailed p < 0.01, *n* = 5). The magnitude of reduction was similar between genotypes (rmANOVA time main effect: f _44.00,308.0_ = 55.69, *p* < 0.0001, rmANOVA genotype main effect: f _1,7_ = 0.5952, *p* = 0.4657, rmANOVA time x genotype interaction: f _44,308_ = 0.5265, *p* = 0.9944). Evoked transients were not measurable with the eCBmut sensor in either DGLα^flx/flx^ and DGLα^D1-Cre+^ mice with AM251 application (Fig. 1i, j; DGLα^flx/flx^ *n* = 4 slices; DGLα^D1-Cre+^ *n* = 4 slices), indicating that the drug doesn’t affect baseline fluorescence associated with the sensor. The sodium channel blocker, TTX, completely blocked evoked eCB transients in DGLα^D1-Cre+^ and DGLα^flx/flx^ eCB2.0 slices (Fig. 1k, j, DGLα^flx/flx^ 5% ± 2%, Mann-Whitney test, two-tailed *p* < 0.05, *n* = 4; DGLα^D1-Cre+^ 12% ± 3%, Mann–Whitney test, two-tailed *p* < 0.001, *n* = 6). The magnitude of TTX blockade was similar between the genotypes (rmANOVA genotype main effect: f _1,8_ = 1.799, *p* = 0.2166). This finding demonstrates that these transients depend on action potential production and ensuing synaptic transmission.

### Ethanol inhibits dMSN 2-AG signaling at corticostriatal synapses

**Fig. 2:**
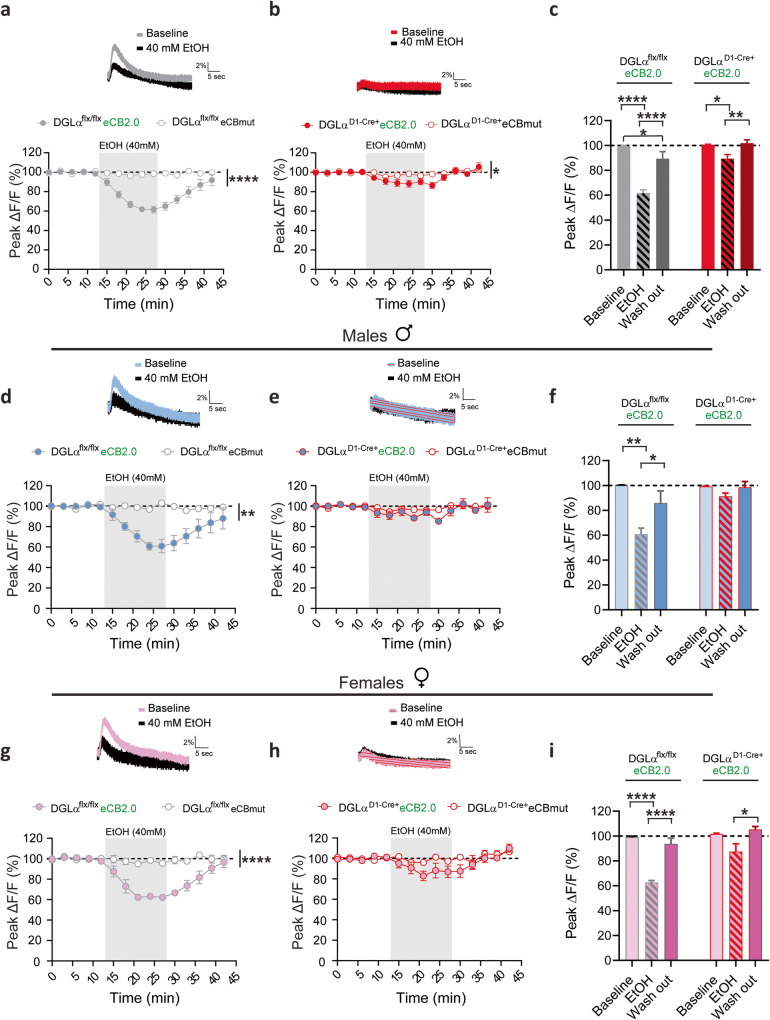
Targeted deletion of dMSN 2-AG signaling dampens ethanol induced eCB inhibition. Inserts: Representative traces of eCB transients from corticostriatal afferents in the DLS at baseline and following ethanol application (gray shaded area) from slices obtained from DGLα ^flx/flx^ (gray trace) or DGLα ^D1 Cre +^ (red trace). Time course of ethanol on eCB transient amplitude evoked by a train of 20 pulse at 20 Hz before and after ethanol application in male and female slices from DGLα ^flx/flx^ (**a**) and DGLα ^D1 Cre +^ (**b**). **c** Summary bar graph of evoked eCB transient peak amplitudes during baseline (9–12 min), ethanol (24–27 min), and washout (39–42 min) calculated as averaged response from DGLα ^flx/flx^ (gray bars) or DGLα ^D1 Cre +^ (red bars). eCB mediated fluorescence changes detected at corticostriatal terminals following ethanol application in male DGLα ^flx/flx^ (**d**) and DGLα ^D1 Cre +^ (**e**) mice. **f** Average eCB fluorescence changes during baseline, ethanol, and ethanol washout periods in male slices expressing eCB2.0 in DGLα ^flx/flx^ (blue and gray bars) and DGLα ^D1 Cre +^ mice (blue and red bars). Changes in eCB-eCB2.0 corticostriatal activation fluorescence in response to ethanol administration in female DGLα ^flx/flx^ (**g**) and DGLα ^D1 Cre +^ (**h**) mice. **i** Summary of eCB2.0 fluorescence data presented in (**g**, **h**). Data reported as mean ± SEM. *p < 0.05, **p < 0.01, ****p < 0.0001.

Bath application of acute ethanol at 40 mM decreased the amplitude of transients in DGLα^flx/flx^ eCB2.0 slices (*n* = 11) compared to eCBmut (*n* = 10) slices obtained from both males and females (Fig. 2a, rmANOVA sensor main effect: F_1,19_ = 75.22, *p* < 0.0001; time x sensor interaction: F_14,266_ = 14.17, p < 0.0001). Ethanol application also induced a depression of the much smaller transients observed in DGLα^D1-Cre+^ eCB2.0 mouse slices compared to eCBmut slices (Fig. 2b, rmANOVA sensor main effect: F_1,16_ = 5.298, *p* = 0.0351; time x sensor interaction: F_14,224_ = 2.00, *p* = 0.0188). The magnitude of the ethanol-induced inhibition of the evoked eCB transients was greater in slices obtained from the DGLα^flx/flx^ mice compared to DGLα^D1-Cre+^ mice (Fig. 2a, b, rmANOVA genotype main effect: F_1,19_ = 36.42, *p* < 0.0001; time x genotype interaction: F_14,226_ = 6.856, *p* < 0.0001). The ethanol-induced inhibition persisted throughout and for several minutes following drug application as indicated by averaging the last two data points (Fig2c, EtOH 62% ± 3%, rm one-way ANOVA treatment main effect: F_2,20_ = 36.79, *p* < 0.0001), but fluorescence transients gradually returned to near-baseline levels after ethanol was washed from the bath (Fig. 2c, Holm-Sidak multiple comparison: washout vs baseline 89% ± 6%, t_20_ = 2.217, *p* < 0.05) in DGLα^flx/flx^ mice. A similar relationship was observed in the DGLα^D1-Cre+^ mice, in which ethanol induced a smaller, reversible inhibition of evoked eCB activity (Fig. 2c, EtOH 89% ± 3%, rm one-way ANOVA treatment main effect: F_2,18_ = 7.44 *p* = 0.0044, Holm-Sidak multiple comparison: washout vs baseline 102% ± 3%, t_18_ = 0.4184, *p* > 0.05).

The group data presented in Fig. 2a, b were separated and analyzed by sex. Ethanol administration decreased eCB transients in eCB2.0 expressing slices (*n* = 5) compared to eCBmut slices (*n* = 6) obtained from DGLα^flx/flx^ male mice (Fig. 2d, rmANOVA sensor main effect: F_1,8_ = 18.38, *p* = 0.0027; time x sensor interaction: F_14,112_ = 4.72, *p* < 0.0001). There were no differences in evoked eCB transients following ethanol administration in DGLα^D1-Cre+^ eCB2.0 male slices compared to eCBmut (Fig. 2e, rmANOVA sensor main effect: F_1,8_ = 2.08, *p* = 0.1875). Ethanol induced a reversible inhibition in DGLα^flx/flx^ eCB2.0 male slices (Fig. 2f, EtOH 61% ± 5%, washout 86% ± 10%, rm one-way ANOVA treatment main effect, F_2,10_ = 12.23, *p* = 0.0021). The ethanol-induced eCB decrease was also observed in DGLα^flx/flx^ slices obtained from female mice compared to female slices expressing the eCBmut sensor (Fig. 2g, *n* = 5/group; rmANOVA sensor main effect: F_1,9_ = 103.8, *p* < 0.0001; time x sensor interaction: F_14,126_ = 10.94, *p* < 0.0001). There was a small, but nonsignificant decrease in evoked eCB transients following ethanol administration in DGLα^D1-Cre+^ eCB2.0 female slices compared to eCBmut slices (Fig. 2h, rmANOVA sensor main effect: F_1,6_ = 2.784, *p* = 0.1463). Ethanol induced a transient decrease in evoked eCB transients in DGLα^flx/flx^ female slices, summarized in Fig. 2i (EtOH 63% ± 2%, washout 94% ± 5%, rm one-way ANOVA treatment main effect, F_2,8_ = 59.03, *p* < 0.0001). There was a significant ethanol-induced decrease in evoked eCB transients in DGLα^D1-Cre+^  female slices summarized in Fig. 2i (EtOH 87% ± 6%, washout 105%  ± 2%, rm one-way ANOVA treatment main effect, F_2,8_ = 5.06, *p* = 0.038). Overall, there were no significant differences between sexes within the genotypes tested (Fig. 2a, b DGLα^flx/flx^ rmANOVA sex main effect: F_1,17_ = 0.014, *p* = 0.9067; DGLα^D1-Cre+^ rmANOVA sex main effect: F_1,14_ = 0.1276, *p* = 0.7262). This study identifies suppression of the mobilization of dMSN-derived 2-AG as a prominent effect of ethanol at primary motor cortex presynaptic terminals in DLS.

### Deletion of dMSN mediated 2-AG signaling decreases ethanol sedative effects

**Fig. 3:**
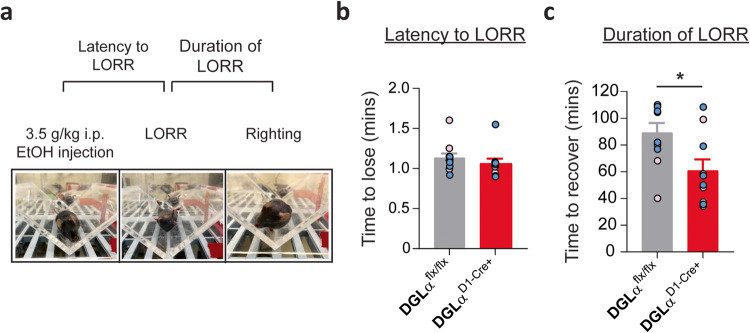
Deletion of striatal dMSN 2-AG signaling reduces duration of loss of righting reflex (LORR). **a** Experimental schematic and pictures of LORR testing paradigm. **b** Latency to lose (**c**) and duration of ethanol induced LORR. Data from individual animals are overlaid as circles, with females in pink and males in blue. DGLα ^flx/flx^ in gray and DGLα ^D1 Cre+^ knockout mice in red. All error bars represent SEM. *t* test, *p < 0.05.

Sensitivity to ethanol’s intoxicating effects has been implicated as a predictor for AUD development risks in humans [44]. In rodents, the sensitivity to the sedative-intoxicating effects of ethanol is assessed using LORR [45, 46]. The latency to and duration of LORR were measured following 3.5 g/kg ethanol administration (Fig. 3a; *n* = 10–11/ group). The latency to LORR did not differ between the DGLα^D1-Cre+^ and DGLα^flx/flx^ mice (Fig. 3b; unpaired two-tailed *t* test: t_19_ = 0.8835, *p* > 0.05). However, the duration of LORR was shorter in the DGLα^D1-Cre+^ compared to DGLα^flx/flx^ mice (Fig. 3c; unpaired two-tailed *t* test: t_19_ = 2.596, *p* < 0.05). Although there was a genotype difference in the duration of LORR, there were no sex differences within the groups (Fig. 3c; *n* = 4–6/ group; DGLα^flx/flx^ unpaired two-tailed *t* test: t_8_ = 0.9577, *p* > 0.05; DGLα^D1-Cre+^ unpaired two-tailed *t* test: t_8_ = 0.6178, *p* > 0.05) or between genotypes (Fig. 3c; females unpaired two-tailed *t* test: t_9_ = 1.623 *p* > 0.05; males unpaired two-tailed *t* test: t_8_ = 2.159 *p* > 0.05) for LORR duration.

### Targeted dMSN 2-AG signaling deletion produces sex-specific ethanol drinking-induced water consumption increase

**Fig. 4:**
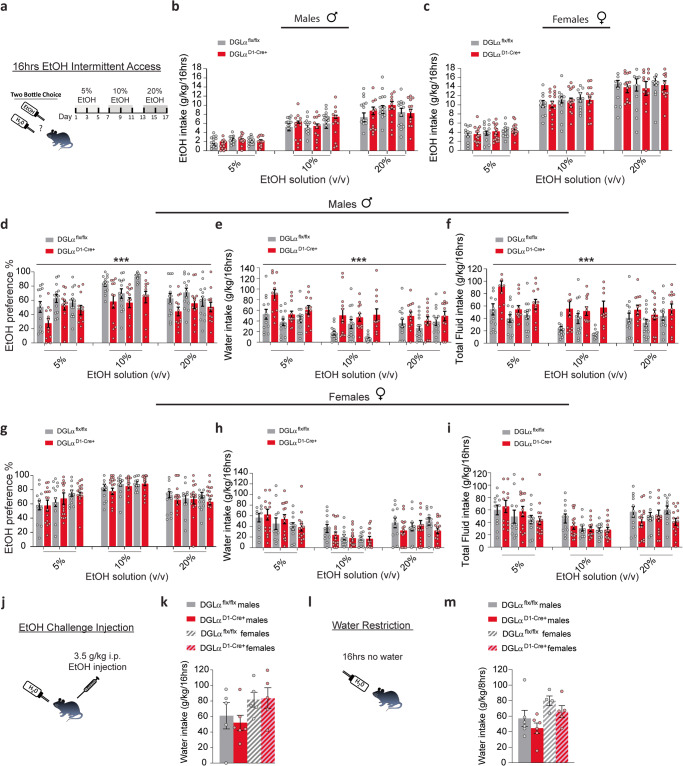
2-AG dMSN signaling deletion results in decreased ethanol preference and increased water consumption in males. Individual data points are overlaid as circles, with DGLα ^flx/flx^ in gray and DGLα ^D1 Cre+^ in red. **a** Schematic of 16 h intermittent ethanol 2-bottle choice drinking paradigm. Ethanol intake in males (**b**) and females (**c**) across 16 h of intermittent drinking. Preference for ethanol, consumption of water, and total fluids in males (**d**–**f**) and females (**g**–**i**) in DGLα ^flx/flx^ and DGLα ^D1 Cre+^ mice. Littermate controls, DGLα ^flx/flx^, represented as gray bars and DGLα ^D1 Cre+^ mice represented as red bars. Individual data points for females shown as pink circles and males as blue circles for each genotype. **j** Schematic of experimental procedure. **k** Water consumption following a challenge injection of ethanol. **l** Schematic of water restriction experimental procedure. **m** Water consumption following 16 h of water restriction. Data presented as mean ± SEM. ***p < 0.001.

Recently, it was shown that global deletion of 2-AG resulted in the reduction of voluntary alcohol consumption [40]. To further assess the role of dMSN 2-AG signaling in voluntary ethanol consumption, we used a two-bottle 16 h intermittent access free-choice protocol. Mice were given a choice of water or escalating concentrations of ethanol overnight for 1wk (Fig. 4a). There were no significant differences in ethanol intake in males (Fig. 4b, *n* = 12–13/ group; rmANOVA, genotype main effect: F_1,69_ = 2.539, *p* = 0.116) or females (Fig. 4c, *n* = 11–15/ group; rmANOVA, genotype main effect: F_1,72_ = 0.487, *p* = 0.487) DGLα^D1-Cre+^ mice compared to DGLα^flx/flx^ mice. However, there was a significant decrease in ethanol preference in the male DGLα^D1-Cre+^ mice compared to DGLα^flx/flx^ mice (Fig. 4d, rmANOVA, genotype main effect: F_1,69_ = 18.369, *p* < 0.001), which is driven in part by day-dependent changes in ethanol preference (rmANOVA, day main effect: F_2,125.5_ = 3.457, *p* = 0.04). The decrease in ethanol preference was accompanied by an increase in water consumption in DGLα^D1-Cre+^ males (Fig. 4e, rmANOVA, genotype main effect: F_1,69_ = 21.752, *p* < 0.001). Water consumption varied across days of testing (rmANOVA, day main effect: F_2,135.6_ = 3.323, *p* = 0.04; rmANOVA, day x genotype interaction: F_2,135.6_ = 1.935, *p* = 0.149). Similar patterns of water consumption were observed in the DGLα^flx/flx^ male and female mice (Fig. 4e, h, rmANOVA, sex main effect: F_1,66_ = 1.972, *p* = 0.165). Moreover, DGLα^D1-Cre+^ males consumed more total fluid compared to DGLα^flx/flx^ males that varied in consumption across days (Fig. 4f, rmANOVA, genotype main effect: F_1,69_ = 22.405, *p* < 0.001; rmANOVA, day main effect: F_2,135.7_ = 3.226, *p* = 0.044; rmANOVA, day x genotype interaction: F_2,135.7_ = 1.991, *p* = 0.141). In females, there was no statistical significance in ethanol preference (Fig. 4g, rmANOVA, genotype main effect: F_1,72_ = 0.563, *p* = 0.455), and there was no difference in water or total fluid intake between the DGLα^D1-Cre+^ and DGLα^flx/flx^ female mice (Fig. 4h, rmANOVA, genotype main effect: F_1,72_ = 0.527, *p* = 0.470; Fig. 4i; rmANOVA, genotype main effect: F_1,72_ = 0.675, *p* = 0.414). Intermittent ethanol access is known to promote a robust escalation in ethanol intake [47, 48]. Indeed, there was a significant increase in ethanol intake in both genotypes as a function of ethanol concentration in males (Fig. 4b, rmANOVA, concentration main effect: F_2,69_ = 82.5, *p* < 0.001) and females (Fig. 4c, rmANOVA, concentration main effect: F_2,72_ = 102.58, *p* < 0.001). Female mice of both genotypes consumed more ethanol than male mice (rmANOVA, sex main effect: F_1,144_ = 26.992, *p* < 0.001).

To determine if increased water intake was driven simply by ethanol exposure, a subset of mice was given a challenge dose of ethanol (3.5 g/kg), and water intake was measured for 16 h (Fig. 4j). There was no significant difference in water intake post ethanol injection in both males and females DGLα^D1-Cre+^ mice compared to DGLα^flx/flx^ mice (Fig. 4k; *n* = 5–6 males/group; unpaired two-tailed *t* test: t_9_ = 0.4828, *p* > 0.05; *n* = 5 females/group; unpaired two-tailed *t* test: t_8_ = 0.115, *p* > 0.05). Furthermore, to determine if water restriction would alter water consumption differently in the DGLα^D1-Cre+^ mice, we water restricted animals overnight and measured water consumption the following day (Fig. 4l). Water intake was similar in both males and females following water restriction (Fig. 4m; *n* = 6 males/group; unpaired two-tailed *t* test: t_10_ = 1.019, *p* > 0.05; *n* = 4–5 females/group; unpaired two-tailed *t* test: t_7_ = 1.356, *p* > 0.05). These findings suggest that the sex-specific water intake increase during two-bottle drinking in the DGLα^D1-Cre+^ mice is context specific, with voluntary ethanol taking but not passive administration (i.p.) of ethanol resulting in increased water consumption.

### Deletion of dMSN 2-AG signaling does not alter consumption of sweet or bitter tastants

**Fig. 5:**
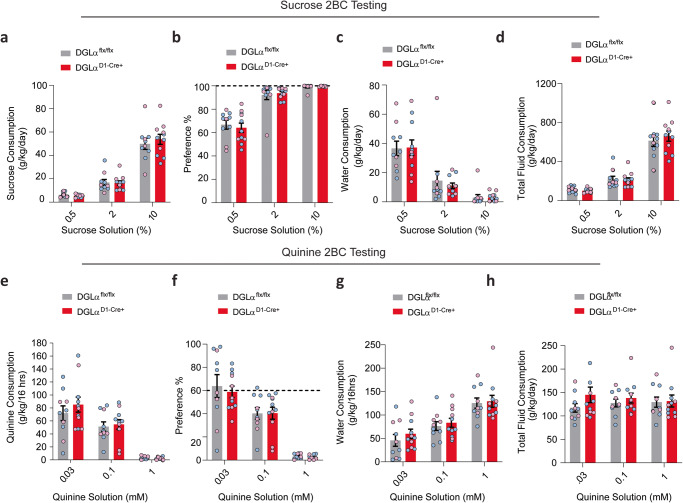
DGLα ^D1 Cre+^ knockout mice display similar taste preference and aversion for sweet- and bitter-flavored solutions. Summary bar graph depicting DGLα ^flx/flx^ mice in gray and DGLα ^D1 Cre+^ mice in red. Individual differences between males (blue circles) and female (pink circles) are shown. **a** Sucrose consumption, (**b**) preference, (**c**) water consumption, and (**d**) total fluid consumption during two bottle choice testing for increasing concentration of sucrose solutions. **e** Quinine consumption, (**f**) preference, (**g**) water consumption, and (**h**) total fluid consumption during quinine two bottle choice testing. All data reported as mean ± SEM.

A subset of mice was administered sucrose and quinine following ethanol exposure to determine the hedonic impact of dMSN mediated 2-AG signaling disruption on taste. Animals were given free choice of increasing concentrations of sucrose (0.5, 2, 10%) or water. Both genotypes showed increased sucrose intake and preference with escalating concentrations (Fig. 5a; *n* = 10–11/ group; rmANOVA, concentration main effect: F_1.239, 21.07 =_ 209.1, *p* < 0.0001; Fig. 5b, rmANOVA, concentration main effect: F_1.147, 19.51_ = 72.72, *p* < 0.0001). DGLα^D1-Cre+^ and DGLα^flx/flx^ mice consumed equal amounts of and showed similar preference for sucrose (Fig. 5a; rmANOVA, genotype main effect: F_1, 17_ = 0.1558, *p* = 0.6980; Fig. 5b, rmANOVA, genotype main effect: F_1, 17_ = 0.1672, *p* = 0.6877). There were no sex differences in sucrose consumed (Fig. 5a; rmANOVA, sex main effect: F_1, 17_  = 1.881, *p* = 0.1881), or sucrose preference between genotypes (Fig. 5b; rmANOVA, sex main effect: F_1,17_ = 0.9960, *p* = 0.3323). Water intake decreased with increasing sucrose concentrations for both genotypes (Fig. 5c, rmANOVA, concentration main effect: F_1.215, 20.65_ = 36.76, *p* < 0.0001). Both DGLα^D1-Cre+^ and DGLα^flx/flx^ mice decreased their water consumption similarly with increasing sucrose concentrations (Fig. 5c, rmANOVA, genotype main effect: F_1,17_  = 0.4966, *p* = 0.4905; rmANOVA, concentration x genotype interaction: F_2,34_ = 0.1937, *p* = 0.8248). No sex difference was observed in water consumption with escalating sucrose concentrations (Fig. 5c; rmANOVA, sex main effect: F_1, 17_ = 2.021, p = 0.1732). Total fluid consumption was similar between the genotypes and sexes (Fig. 5d, rmANOVA, genotype main effect: F_1,17_ = 0.1088, *p* = 0.7456, rmANOVA, sex main effect: F_1,17_ = 2.235, *p* = 0.1532).

Following sucrose preference testing, mice were given access to water or escalating quinine concentrations (in Mm, 0.03, 0.1, 1). No differences in consumption of quinine between DGLα^flx/flx^ and DGLα^D1-Cre+^ of both sexes were found (Fig. 5e, rmANOVA, genotype main effect: F_1, 17_ = 0.3296, *p* = 0.5734, rmANOVA, sex main effect: F_1, 17_  = 0.8833, *p* = 0.3605). Both genotypes decreased quinine consumption with increased concentrations (Fig. 5e, rmANOVA, concentration main effect: F_1.497, 25.45_ = 61.34, *p* < 0.0001, rmANOVA, concentration x genotypes interaction: F_2, 34_ = 0.6281, *p* = 0.5397). Similarly, there were no differences in quinine preference between genotypes (Fig. 5f, rmANOVA, genotype main effect: F_1, 17_  = 0.1114, *p* = 0.7426). Both males and females decreased their quinine preference in a similar manner as the concentration increased (Fig. 5f, rmANOVA, sex main effect: F_1, 17_  = 2.507, *p* = 0.1317, rmANOVA, concentration main effect: F_1.589, 27.01_  = 77.57, *p* < 0.0001, rmANOVA, sex x concentration interaction: F_2, 34_  = 1.217, *p* = 0.3087).Water intake did not differ significantly between genotypes or sexes (Fig. 5g, rmANOVA, genotype main effect: F_1, 17_ = 0.3409, *p* = 0.5670, rmANOVA, sex main effect: F_1, 17_ = 2.282, *p* = 0.1493). Likewise, we did not observe differences in total fluid intake between genotypes or sexes (Fig. 5h, rmANOVA, genotype main effect: F_1, 17_ = 0.5629, *p* = 0.4633, rmANOVA, sex main effect: F_1, 17_ = 0.5360, *p* = 0.4741). The preferences and consumption for nonalcohol tastants were not altered with dMSN DGLα deletion.

## Discussion

We assessed the effects of targeted deletion of DGLα, the 2-AG biosynthesis enzyme, in striatal dMSNs on presynaptic eCB signaling and ethanol effects on this signaling and behavior. We first used the GRAB_eCB2.0_ biosensor to demonstrate that real-time eCB mobilization that affects corticostriatal presynaptic terminals is reduced in dMSN DGLα KO mouse striatum in response to single and burst stimuli. These findings support a role for dMSN 2-AG signaling and are consistent with previous findings that such stimulation induces mainly 2-AG mobilization from the entire MSN population that affects corticostriatal synapses [42]. Interestingly, the decrease in eCB signaling in the dMSN DGLα KO mouse slices appears to be greater than might be expected if both d- and iMSNs contribute equally to stimulus-induced 2-AG production. However, the contribution of other neuronal eCB sources, as well as residual signaling from other 2-AG biosynthesis pathways or eCBs, such as AEA, remains unclear [49]. It is also important to mention that 2-AG levels are reduced in the dMSN DGLα KO mice [41]. Both 2-AG and AEA have been implicated in striatal plasticity, particularly LTD [30, 50–53]. However, most of the evidence suggests a role of AEA in LTD [30, 50, 51]. Thus, our findings and those of Liput and coworkers [42], are surprising. However, LTD is generally induced by stimuli with frequencies and durations greater than those used in the present study and by Liput and coworkers [42]. The corticostriatal afferents are an important site of eCB signaling in the striatum [3, 54–58], and thus this role for dMSN-derived 2-AG has important implications for striatal function and related behaviors. However, it is worth mentioning that eCBs modulate synaptic functions at GABAergic striatal synapses which may be even more sensitive than corticostriatal synapses [59–62].

Our findings also demonstrate that ethanol induces transient alterations in 2-AG mobilization at corticostriatal afferents. Furthermore, we showed that the selective deletion of DGLα from dMSNs resulted in dampening of ethanol effects on eCB signaling at these terminals. These findings indicate that acute ethanol exposure dampens retrograde eCB signaling involved in synaptic depression, most likely via inhibition of eCB production or release. Endocannabinoid modulation is thought to contribute to compulsive drug seeking and taking behaviors, including those induced by ethanol [9, 63, 64]. Endocannabinoid subtypes like 2-AG might have differential roles in different brain regions and reward-driven behaviors [3, 39]. The present data show that acute ethanol exposure reduces striatal 2-AG release/CB1 receptor activation in the dorsolateral striatum at corticostriatal terminals. Previous reports have shown that 2-AG levels increase in the nucleus accumbens during ethanol self-administration in vivo [39]. Our data do not contradict previous findings but highlight and further support the hypothesis that eCB subtypes may mediate different signaling profiles in different brain areas. It will be interesting to determine the contribution of ethanol inhibition of 2-AG signaling at specific corticostriatal synapses to ethanol-related behaviors. Also, more research is needed to determine whether ethanol induces differential eCB signaling in response to acute and repeated drug exposure.

Although the newly developed eCB sensor has different affinities for 2-AG and AEA, the sensor can detect both eCBs [43]. Given that the sensor has a lower affinity for 2-AG, it would be difficult to detect very low levels of 2-AG signaling. However, our work confirms previous findings from our group that showed eCB transients evoked by brief stimuli detected at these corticostriatal afferents arising from primary motor cortex are primarily 2-AG mediated [42]. Together, our findings further support our hypothesis that 2-AG and AEA may have differential functions in different brain regions at different synapses that may be dependent on sensory input and/or external stimuli (e.g., electrical stimulation) [3]. In the next generation of eCB sensors, new variants with improved dynamic range and eCB type specificity are needed to fully understand the contributions of eCB subtypes to synaptic signaling and behavior.

The eCB system is altered by acute and chronic ethanol exposure [22, 65]. Although the CB1 receptor role in ethanol’s rewarding properties has been extensively studied, the roles of 2-AG and AEA in these processes are not fully understood [26, 27, 40, 66–68]. Ethanol self-administration increases striatal 2-AG levels, without accompanying changes in AEA levels [39]. These findings suggest that 2-AG may play a significant role in the reinforcing effects of ethanol. This is further supported by a reduction in ethanol consumption in global 2-AG deficient mice [40]. The present data reveal that ethanol inhibits 2-AG signaling predominantly at terminals innervating dMSNs in the dorsolateral striatum, as shown in Fig. 2. CB1 receptor-2-AG activation has the potential to reduce synaptic activation of dMSNs. This in turn, can reduce striatal GABAergic transmission in output brain regions, such as the substantia nigra pars compacta (SNc) and pars reticulata (SNr). For example, decreased inhibitory transmission in the SNc may result in the disinhibition of dopamine neurons, such that dMSN-mediated 2-AG-CB1 receptor activation can contribute to ethanol consumption by enabling striatal dopamine release [69, 70]. Alternatively, the loss of ethanol-induced eCB modulation of dMSN synapses may result in the enhancement of dMSN roles in behavior through the disinhibition of dorsolateral striatal synaptic activity. This hypothesis is supported by previous work that showed a strengthening of dMSN function after ethanol exposure [35].

Previous studies suggest that chronic in vivo exposure to misused substances, including alcohol, results in predominate changes in synaptic transmission and anatomical properties involving dMSNs [35, 65]. Our findings suggest that most of the ethanol-induced 2-AG inhibition is mediated by dMSNs (Fig. 2). However, this study cannot unequivocally rule out the contributions of iMSN 2-AG signaling to the ethanol induced inhibition observed in Fig. 2. Therefore, more research on the role of iMSN 2-AG signaling in ethanol effects on transmission and behavior is needed to further understand the cell-specific mechanisms involved in the effects of ethanol on the eCB system.

Ethanol induced alteration in striatal 2-AG signaling in the dMSN DGLα KO mice is accompanied by a reduction in the sedative/hypnotic effects of ethanol (Fig. 3). This finding could suggest either a change in sensitivity to ethanol or a change in ethanol metabolism that results in faster reductions in blood ethanol concentration leading to faster regain of righting reflex in the dMSN DGLα KO mice. A metabolic change seems unlikely given the neuronal targeting of the enzyme knockout. Nonetheless, future experiments examining blood ethanol concentration can assess possible changes in metabolism. This finding differs from previous experiments in the global CB1 knockout mouse indicating increased ethanol-induced sedation [32] an effect that likely involves synapses other than the corticostriatal inputs to dMSNs. Genetic background can also influence ethanol-induced behavioral responses, such as sedation [71].

Additionally, the eCB system has been implicated in the development of habitual behaviors, as well as the voluntary taking of ethanol [12, 72, 73]. It was previously unclear whether ethanol can induce differential eCB responses involved in different ethanol-related behaviors. Our findings extend a previous report that indicates involvement of 2-AG signaling in voluntary ethanol consumption [40]. Deletion of DGLα from dMSNs decreased ethanol preference in a voluntary intake paradigm in males but not females. This change was not accompanied by a decrease in ethanol intake, but rather an increase in water consumption and total fluid intake in males. Ethanol intake varied depending on the day/session in which the ethanol concentration was administered. However, the day-dependent changes in ethanol consumption accounts for ~5% of the variance in the data. It is possible that loss of 2-AG signaling at cortical inputs to dMSNs increases fluid intake, but this effect is counteracted in the presence of ethanol due to taste or the neural effects of the drug. However, increased water intake was not seen with ethanol exposure via intraperitoneal injection, nor when ethanol was exchanged for sucrose or quinine. Thus, we find no evidence that ethanol intoxication per se or the presence of preferred or non-preferred tastants drives increased water intake in the dMSN DGLα KO mice. Thus, there is something specific about voluntary ethanol ingestion that drives up water intake in these mice, perhaps due to the unique taste of ethanol or presence of mild neural effects of ethanol that induce greater thirst and cannot be mimicked by intraperitoneal drug injection. Although our findings suggest that ethanol exposure and 2-AG signaling may promote and/or facilitate ethanol preference via eCB depression of transmission onto dMSNs, other paradigms of drinking, such as ethanol-primed and cue-induced ethanol-seeking behavior should be investigated to further evaluate possible 2-AG eCB effects on voluntary ethanol taking behaviors.

Although our cell-type specific deletion of 2-AG signaling in corticostriatal synapses onto dMSNs in DLS may suggest a role for this signaling in the development of habitual behavior, this is likely not the only mechanism by which ethanol exerts its function. Undoubtedly, 2-AG signaling at other synapses within different brain regions may contribute to alcohol-related behaviors. Nucleus accumbens 2-AG levels are increased in vivo during ethanol self-administration [39]. Therefore, dMSN 2-AG signaling deficits in this area likely also contribute to ethanol-related behaviors. Furthermore, prefrontal cortex has been implicated in the modulation of ethanol seeking and taking behaviors [74–77]. It is likely that 2-AG signaling deficits in the cortex may alter glutamatergic output in subcortical areas, thereby altering the motivational salience of ethanol [78]. Drug salience is important for the establishment of drug dependence [79]. For example, if cortical drive is diminished due to deficits in 2-AG signaling, this may result in the perceived decrease in ethanol’s rewarding effects.

Our work has highlighted that eCB signaling mechanisms at specific synapses and release from specific neurons can have profound signaling and behavioral effects produced by ethanol. Moreover, our findings provide a new potential therapeutic target research avenue of exploration, involving dMSN-mediated 2-AG signaling and direct pathway function for treating AUD.
